# Relationship between Postural Deformities and Frontal Function in Parkinson's Disease

**DOI:** 10.1155/2015/462143

**Published:** 2015-08-13

**Authors:** Satoko Ninomiya, Akihiko Morita, Hiroko Teramoto, Takayoshi Akimoto, Hiroshi Shiota, Satoshi Kamei

**Affiliations:** Division of Neurology, Department of Medicine, Nihon University School of Medicine, 30-1 Oyaguchi Kami-cho, Itabashi-ku, Tokyo 173-8610, Japan

## Abstract

Postural deformities and executive dysfunction (ED) are common symptoms of Parkinson's disease (PD); however, the relationship between postural deformities and ED in patients with PD remains unclear. This study assessed the relationship between postural deformities and ED in patients with PD. Sixty-five patients with sporadic PD were assessed for the severity of postural deformities and executive function. The severity of postural deformities was scored using the United Parkinson's Disease Rating Scale item 28 score: no postural deformity (0), mild postural deformities (1), or severe postural deformities (2–4). Executive function was assessed using the Behavioral Assessment of the Dysexecutive Syndrome (BADS) and an age-controlled standardized BADS score <70 was defined as ED. Age-controlled standardized BADS scores were compared across the three groups using the Kruskal-Wallis test. Relationship between ED and the severity of postural deformities was assessed using the Mann-Whitney *U* test. Age-controlled standardized BADS score significantly differed among the three groups (*P* = 0.005). ED was significantly related to the severity of postural deformities (*P* = 0.0005). The severity of postural deformities was associated with a lower age-controlled standardized BADS score and ED, and these findings suggest that postural deformities were associated with frontal dysfunction in patients with PD.

## 1. Introduction

Parkinson's disease (PD) is a chronic neurodegenerative disease characterized by motor symptoms such as akinesia, rigidity, resting tremor and postural abnormalities, and nonmotor symptoms including dementia, depression, and executive dysfunction (ED) [1]. These symptoms have a major negative impact on the quality of life of patients with PD [2]. Postural abnormalities in patients with PD include deformities and instability [3]. Postural deformities such as stooped posture, camptocormia, anterocollis, dropped head syndrome, Pisa syndrome, and scoliosis induce clinical impairment at the late stage of PD [3, 4]. Postural instability is due to dysfunctional postural reflexes and causes falls and gait disturbances [3]. Previous studies have reported that postural deformities in patients with PD were caused by dystonia, rigidity, impaired proprioception, and kinesthesia; however, the underlying pathophysiology of postural deformities in patients with PD is unknown [4]. Some studies have suggested that postural instability and gait disturbance significantly correlate with ED [5, 6]. However, the relationship between postural deformities and ED in patients with PD has not been determined. The Behavioural Assessment of the Dysexecutive Syndrome (BADS) [7] is a neuropsychological battery that is used to assess ED with ecological validity and it is sensitive to ED in PD patients [8]. We previously evaluated the relationship between the freezing of gait and ED using the BADS in patients with PD [9]. The present study assessed the relationship between postural deformities and ED in patients with PD.

## 2. Patients and Methods

### 2.1. Patients

Consecutive patients who were diagnosed with sporadic PD according to the United Kingdom Parkinson's Disease Brain Bank criteria [10] at the Neurology Clinic, Nihon University Itabashi Hospital, between December 2006 and October 2008, were enrolled. Patients diagnosed with other forms of parkinsonism such as dementia with Lewy bodies [11, 12], drug-induced parkinsonism, vascular parkinsonism, and atypical parkinsonism with absent or minimal responses to antiparkinsonian drugs were excluded. All patients were assessed using cranial magnetic resonance imaging and those with intracerebral ischemic changes including a single asymptomatic lacuna or slight periventricular hyperintensity according to the reported classification of periventricular hyperintensity [13] were excluded. All patients were assessed using the United Parkinson's Disease Rating Scale (UPDRS) [14] and the Mini-Mental State Examination (MMSE) based on the* Diagnostic and Statistical Manual of Mental Disorders, 4th edition* (DSM-IV) [15]. Executive function was assessed using the BADS [16]. ED was defined as an age-controlled standardized BADS score <70 [7, 16]. Informed written consent for participation in the present study was obtained from each patient according to a protocol approved by Institutional Research Review Board of Nihon University.

### 2.2. Assessment of Postural Deformities

The severity of postural deformities was assessed using UPDRS item 28 score, which classified patients into five grades according to severity: (0) normal erect, (1) not quite erect, slightly stooped posture: it could be normal for older person, (2) moderately stooped posture, definitely abnormal: it can be slightly leaning to one side, (3) severely stooped posture with kyphosis: it can be moderately leaning to one side, and (4) marked flexion with extreme abnormality of posture. The patients were classified into three groups according to UPDRS item 28 score: no postural deformity (score of 0), mild postural deformities (score of 1), or severe postural deformities (score of 2–4).

### 2.3. Statistical Analysis

All data were statistically analyzed using IBM SSPS statistics for Windows version 22 (IBM Corp, Armonk, NY). The Shapiro-Wilk normality test was employed to evaluate whether continuous variables exhibited a normal distribution. Parametric analysis was applied to normal data and nonparametric analysis was applied to nonnormal data. All continuous variables, including age at the time of assessment, duration of disease from onset, Hoehn and Yahr (HY) stage, UPDRS total score, MMSE score, and age-controlled standardized BADS score, are expressed as median (minimum, maximum). The categorical variable of male/female ratio is expressed as a percentage. Differences in continuous variables across the three groups (no postural deformity, mild postural deformities, and severe postural deformities) were assessed using the Kruskal-Wallis test. The difference in the male/female ratio across the three groups was assessed using Fisher's exact test. The relationship between age-controlled standardized BADS score and the severity of postural deformities in patients with PD was assessed using the Kruskal-Wallis test, and when statistical significance was revealed,* post hoc* tests were performed with the following combinations of groups: no postural deformity versus mild postural deformities, no postural deformity versus severe postural deformities, and mild postural deformities versus severe postural deformities. The relationship between the severity of postural deformities and ED was assessed using the Mann-Whitney *U* test. The level of statistical significance for all tests in this study was defined as 0.05.

## 3. Results

One hundred and forty-eight consecutive patients diagnosed with the sporadic form of PD were registered during the study period, and 65 of these were confirmed as sporadic PD and appropriately assessed using UPDRS and BADS and were enrolled in the study. The characteristics of this sample of PD patients are described elsewhere [17].

Table 1 shows the demographic characteristics of the three groups of patients with PD. The results of the Kruskal-Wallis test revealed that age at assessment, disease duration, HY stage, and UPDRS total score significantly differed among the three groups. Patients with more severe postural deformities had an older age, a longer duration of disease, a higher HY stage, and a greater UPDRS total score. The male/female ratio and the MMSE score did not significantly differ among the three groups.

Table 2 shows that the median age-controlled standardized BADS score for patients with PD who had no postural deformity or mild postural deformities was ≥70 (without ED) and that for those with severe postural deformities was <70 (with ED). The age-controlled standardized BADS score significantly differed among the three groups (*P* = 0.005, Kruskal-Wallis test).* Post hoc* tests revealed a significant difference in age-controlled standardized BADS score between PD patients with no postural deformity and PD patients with severe postural deformities (*P* = 0.004).

Table 3 shows the relationship between the severity of postural deformities in PD patients and ED. Postural deformities were significantly more severe in patients with ED (*P* = 0.0005; Mann-Whitney *U* test).

## 4. Discussion

One-third of all patients with PD develop postural deformities [18]. The more severe postural deformities lead to back pain, difficulties with walking, breathlessness, unsteadiness leading to falls [4], and impaired activities of daily life [3, 4, 18]. Dystonia, rigidity, impaired proprioception, and kinesthesia were previously thought to cause postural deformities [4]; however, they do not adequately explain the mechanisms of postural deformities. Deep brain stimulation of the pallidum to treat camptocormia resulted in no to modest improvement [4, 19], and such postural deformities are generally considered to be levodopa nonresponsive [3, 4]. Pisa syndrome associated with Alzheimer's disease has been treated with cholinesterase inhibitors [4].

The relationship between ED in patients with PD and motor symptoms such as falls and pulsion was identified [20, 21]. We previously revealed a relationship between freezing of gait and ED in patients with PD [9], and here we discovered a relationship between postural deformities and ED evaluated by the BADS. The BADS is an extensive neurological battery composed of six subtests that evaluate executive functions including set shifting, inhibition control, planning, problem solving, and reasoning [7]. Executive function is mediated predominantly by the frontal lobe [22, 23]. Although the MMSE evaluates global cognitive performance such as orientation, registration and short-term recall, attention and concentration, language, and visuospatial function [15], it does not sufficiently measure executive functions such as reasoning, planning, and set shifting [24]. Camptocormia and abnormal neck posture such as anterocollis were not reported to be related to MMSE score in patients with PD [25–27] and this study also did not show an association between MMSE score and postural deformities in patients with PD.

In the present study, patients with PD were classified into three groups according to the severity of postural deformities defined using UPDRS item 28 score. Those with a score of 1 (mild postural deformities) had stooped posture, whereas those with a score of 2–4 (severe postural deformities) had stooped posture and scoliosis [14], which are very common in the later stages of PD [4]. The results of this study suggest that scoliosis was significantly associated with frontal dysfunction.

This study also identified a relationship between severe postural deformities and more advanced age and longer disease duration and higher HY stage, indicating an association between the progression of PD and postural deformities. A previous study reported that patients with camptocormia had an older age, a longer disease duration, and a higher degree of severity assessed according to HY stage and UPDRS total score [28]. Another study showed that age and disease duration correlated with the severity of anterior and lateral flexion, and dropped head was associated with a higher HY stage [29].

The pathophysiological mechanism of postural deformities in PD is unclear. Dysfunction of the basal ganglia-brainstem system and the corticostriatal circuits may be associated with the pathogenesis of motor disturbances in PD [30]. Frontal executive functions are correlated with motor function. Frontal-subcortical circuits that connect the frontal lobe and basal ganglia mediate many aspects of behavior and cognition [31]. ED is related to dysfunction within the frontostriatal circuits. Correlations between ED and motor symptoms including freezing of gait [9] and postural instability [32] were caused by common pathophysiological mechanisms linked to frontal-subcortical circuits. In our study, postural deformities correlated with frontal-executive function. This might be due to impairment of the cerebral cortex and the basal ganglia [33]. Postural deformities and cognitive functions are therefore considered to share a common pathophysiology.

## 5. Conclusion

This study is the first to assess the relationship between postural deformities and frontal function in patients with PD. The severity of postural deformities was significantly associated with frontal function in patients with PD.

## Figures and Tables

**Table 1:** Demographics and disease characteristics of the patients.

	No postural deformity	Mild postural deformities	Severe postural deformities	*P*
*n*	20	30	9	

Age, years	56.5 (34, 76)	71 (50, 84)	71.5 (57, 84)	<0.001^*∗*^
Male	12 (60%)	14 (42%)	5 (42%)	0.421
Disease duration, months	60 (12, 228)	57 (1, 138)	89 (36, 216)	0.017^*∗*^
HY stage	2 (1, 2)	3 (2, 3)	3.5 (2, 5)	<0.001^*∗*^
UPDRS total score	24 (13, 41)	38 (20, 61)	57.5 (30, 111)	<0.001^*∗*^
MMSE score	29 (24, 30)	26.5 (19, 30)	25.5 (6, 30)	0.087

Data are expressed as median (minimum, maximum) or *n* (%).

HY: Hoehn and Yahr; *n*: number of patients; MMSE: Mini-Mental State Examination; UPDRS: Unified Parkinson's Disease Rating Scale.

UPDRS item 28 score of 0, 1, and 2–4 were used to define no postural deformity, mild postural deformities, and severe postural deformities, respectively.

*P* values were calculated using the Kruskal-Wallis test or Fisher's exact test as appropriate.

^*∗*^Statistically significant (*P* < 0.05).

**Table 2:** Relationship between age-adjusted standardized BADS score and severity of postural deformities in patients with PD.

	*n*	Age-adjusted standardized BADS score	*P*
No postural deformity	20	97.5 (54, 123)	0.005^*∗*^
Mild postural deformities	30	76.5 (34, 124)	
Severe postural deformities	9	64.0 (19, 109)	
*Post hoc *tests			
No postural deformity versus mild postural deformities			0.200
Mild postural deformities versus severe postural deformities			0.134
No postural deformity versus severe postural deformities			0.004^*∗*^

Data are expressed as median (minimum, maximum).

BADS: Behavioral Assessment of the Dysexecutive Syndrome; *n*: number of patients; UPDRS: Unified Parkinson's Disease Rating Scale.

UPDRS item 28 score of 0, 1, and 2–4 were used to define no postural deformity, mild postural deformities, and severe postural deformities, respectively.

*P* values were calculated using the Kruskal-Wallis test as appropriate.

*Post hoc *tests were performed to determine the significance of differences between the following combinations of PD groups: no postural deformity versus mild postural deformities, mild postural deformities versus severe postural deformities, and no postural deformity verus severe postural deformities.

^*∗*^Statistically significant (*P* < 0.05).

**Table 3:** Severity of postural deformities in PD patients with and without ED.

	With ED	Without ED
No postural deformity	1	19
Mild postural deformities	12	18
Severe postural deformities	6	3

ED: executive dysfunction; UPDRS: Unified Parkinson's Disease Rating Scale.

ED was defined as age-controlled standardized score <70 on the Behavioral Assessment of the Dysexecutive Syndrome battery.

UPDRS item 28 score of 0, 1, and 2–4 were used to define no postural deformity, mild postural deformities, and severe postural deformities, respectively.

Date were analyzed using the Mann-Whitney *U* test.

Postural deformities were significantly more severe in PD patients with ED (*P* = 0.0005).
